# Industry responses to unhealthy food retail promotion restrictions: a thematic analysis of two public consultations in Scotland

**DOI:** 10.1017/S1368980025100761

**Published:** 2025-07-18

**Authors:** Clara Gomez-Donoso, Sadika Akhter, Adrian J Cameron, Jean Adams, Martin White, Gary Sacks, Anna Peeters, Kathryn Backholer

**Affiliations:** 1 Institute for Health Transformation, Global Centre for Preventive Health and Nutrition, School of Health and Social Development, Faculty of Health, Deakin University, Geelong, VIC, Australia; 2 MRC Epidemiology Unit, School of Clinical Medicine, University of Cambridge, Cambridge, UK

**Keywords:** Food policy, Retail food environment, Commercial determinants of health, Industry playbook

## Abstract

**Objective:**

Governments are increasingly implementing policies to improve population diets, despite food industry resistance to regulation that may reduce their profits from sales of unhealthy foods. However, retail food environments remain an important target for policy action. This study analysed publicly available responses of industry actors to two public consultations on regulatory options for restricting unhealthy food price and placement promotions in retail outlets in Scotland.

**Design:**

We conducted a qualitative content analysis guided by the Policy Dystopia Model to identify the discursive (argument-based) and instrumental (tactic-based) strategies used by industry actors to counter the proposed food retail policies.

**Setting:**

Scotland, UK, 2017–2019.

**Participants:**

N/A.

**Results:**

Most food and retail industry responses opposed the policy proposals. Discursive strategies employed by these actors commonly highlighted the potential costs to the economy, their industries and the public in the context of a financial crisis and disputed the potential health benefits of the proposals. They claimed that existing efforts to improve population diets, such as nutritional reformulation, would be undermined. Instrumental strategies included using unsubstantiated and misleading claims, building a coordinated narrative focused on key opposing arguments and seeking further involvement in policy decision-making.

**Conclusions:**

These findings can be used by public health actors to anticipate and prepare for industry opposition when developing policies targeted at reducing the promotion of unhealthy food in retail settings. Government action should ensure robust management of conflicts of interest and establishment of guidance for the use of supporting evidence as part of the public health policy process.

Unhealthy diets with excessive consumption of highly processed food and drinks that are high in added sugar, salt and/or saturated fat are a leading risk factor for non-communicable diseases^(1,2)^. Retail food environments serve as the interface between individuals and the food system^(3)^. As a result, marketing strategies in retail settings play a crucial role in shaping the availability, affordability and desirability of food, which in turn influences purchasing behaviours^(4)^. Marketing strategies, which are influenced by manufacturers and retailers, include discounted prices, highly visible displays and other promotional practices^(3,4)^. The disproportionate promotion of less healthy foods in retail settings may undermine ongoing efforts to promote healthy diets, including the effectiveness of public health policies such as sugar-sweetened beverage (SSB) taxes^(5,6)^.

In United Kingdom supermarkets, more than 80 % of display space at checkouts was occupied by unhealthy food in 2018^(7)^. Similarly, in Australia, leading supermarket retailers allocated over 80 % of ends-of-aisle space to price promoted food and drinks, and price promoted food at checkouts were 7·5 times more likely to be unhealthy than healthy in 2019^(8)^. In the United Kingdom, purchases of price promoted products are skewed towards unhealthy food categories^(9–11)^, with around 40–60 % of unhealthy food purchased on price promotion compared with 20–25 % for healthy food^(11)^. Price promotions can generate substantial short-term increases in sales and consumption of promoted products, and have been shown to impact long-term purchasing patterns^(12,13)^. Placement of products in premium store locations (i.e. in checkout, end of aisle and freestanding displays) can also affect purchasing and dietary behaviour^(14)^.

In 2022, England enacted mandatory restrictions on the marketing of products high in fat, sugar or salt (HFSS) through price and placement promotion strategies in food retail outlets^(15)^. This represented the first time globally that retail promotions appeared on national policy agendas as a means of improving population diets. While placement restrictions were implemented in 2022, the implementation of volume-based price promotion restrictions has been repeatedly delayed based on the alleged burden on businesses and customers amid a cost-of-living crisis^(16)^. Public health commentators have criticised the delay as short-sighted, as it shifts responsibility for non-communicable diseases and dietary choices back to individuals^(17)^. Scotland has proposed similar regulatory action, with the government conducting multiple consultations since 2017 to inform the development of a policy to restrict retail promotions on unhealthy food and drinks^(18–21)^. The most recent consultation^(21)^ closed in May 2024, with a response from the Scottish government still pending at the time of writing.

One of the most frequently cited barriers to food environment policy development and implementation is industry resistance^(22)^. Increasingly powerful transnational corporations exert their structural, instrumental and discursive powers through lobbying, using their market dominance to set policy agendas and framing problems and policy solutions to align with their interests^(23,24)^. A growing body of evidence describes the instrumental and discursive strategies used by the food industry to counter public health policies^(25–27)^. These strategies tend to downplay the potential public health benefits of policies while exaggerating the potential economic and societal costs. Similar strategies are repeatedly observed across transnational corporations providing so-called unhealthy commodities or services, such as tobacco, alcohol, gambling and unhealthy food, to block, weaken and delay public health policies in order to protect their interests^(23)^. Despite their broad similarities, the specific content of industry arguments and the approach to deploying instrumental strategies varies with each policy context.

No study to date has conducted a theoretically guided analysis to document discursive (argument-based) and instrumental (tactic-based) strategies in response to a regulatory proposal restricting unhealthy food and beverage price and placement promotions in food retail settings. This study aimed to identify the arguments and actions used by industry actors in response to two public consultations (2017–2019) on unhealthy food retail promotion restrictions in Scotland.

## Methods

### Study design

We used a theoretically guided qualitative study design, based on an existing model of corporate political activity, the Policy Dystopia Model (PDM)^(28)^, as informed by an initial inductive analysis of a sub-sample of responses submitted by industry actors. A deductive content analysis was undertaken to identify the strategies used by industry actors based on the PDM. Ethical approval was not required as the study only used data already in the public domain.

### Theoretical framework

The PDM was originally developed to examine tobacco industry political activity^(28)^ and has since been adapted to different industries including the food industry^(25–27)^. The PDM describes two types of strategies to influence policy and regulation: (1) discursive strategies include the use of arguments that expand or create potential costs and downplay or deny potential benefits of the proposed policies and (2) instrumental strategies reflect the approach, actions or tactics used to deliver those arguments including coalition management, information management and direct involvement and influence in the policy making process (Table 1).

**Table 1.** Policy dystopia model: discursive and instrumental strategies

Discursive strategies	
Expand or create unanticipated costs to the economy and society	Exaggerating potential negative consequences of the policy on the economy and parts of society
Expand or create unintended costs to public health	Warning that the policy may have unintended negative consequences on public health
Contain or deny the intended public health benefits	Claiming the proposed policy is unlikely to have the intended public health benefits
Contain or deny the expected costs to industry	Downplaying direct potential costs to own industry while emphasising cost to other, more deserving groups such as small businesses
Instrumental strategies	
Coalition management	Building or managing alliances with other companies or societal actors to establish alternative platforms for arguments
Information management	Producing and disseminating industry-favourable information while suppressing and undermining information in support of the policy
Direct involvement and influence in policy	Access to, and representation or involvement in the policy process, including direct lobbying of policymakers
Legal strategies	Legal action or the threat thereof

Reproduced from: Lauber K, Hunt D, Gilmore AB, Rutter H. Corporate political activity in the context of unhealthy food advertising restrictions across Transport for London: A qualitative case study. PLoS Medicine. 2021;18(9):e100369.

### Data collection

Publicly available industry-led submissions in response to two consultations on proposals to restrict price and placement promotions of HFSS products were downloaded from the Scottish Government website in 2023^(29,30)^. Publicly available industry responses represented around 70 % of all industry responses submitted for each consultation.

The first consultation was conducted in 2017/18 (hereafter referred to as ‘consultation 1’ or ‘c1’)^(19)^. Consultation 1 considered a broad variety of policy options to transform the food environment and for healthier and more active lives, including restrictions on unhealthy food price promotions. Responses to consultation 1 were submitted by 179 individuals, 140 public and third sector organisations including health professional and advocacy groups (95 % consented to make their submission publicly available) and 43 industry organisations (70 % publicly available). The second consultation took place in 2018/19 (‘consultation 2’ or ‘c2’)^(20)^. Consultation 2 was specific to price and placement promotion restrictions, focusing on policy design and implementation details. Responses to consultation 2 were submitted by 632 individuals, 55 public and third sector organisations (96 % publicly available) and 39 industry organisations (69 % publicly available). Table 2 shows a summary of the two consultations’ objectives and proposals in relation to restricting food retail promotions.

**Table 2.** Proposals and objectives from consultations related to food retail promotion restrictions of HFSS products (high in fat, sugar and salt). Scottish Government, 2017–2019

Consultation	Proposals and objectives
Consultation 1 ‘A Healthier Future – Action and Ambitions on Diet, Activity and Healthy Weight’ 2017/18	First consideration to restrict promotions on food and drink products which are high in fat, salt and sugar (HFSS) in retail settings. Proposals included price promotion restrictions on multi-buys (e.g. X for £Y) and temporary price reductions (e.g. % or £X off). Suggested approaches for selecting targeted products included the consideration of a) the existing nutrient profiling model developed by the Food Standards Agency, b) the content of a specific nutrient (e.g. sugar) or c) foods that contribute the most calories to the diet. Consultation invited views on types of promotions considered and definitions for the types of food and drink subject to restrictions.
Consultation 2 ‘Reducing health harms of foods high in fat, sugar or salt’ 2018/19	Follow-up consultation considering price (multi-buys and sales of unlimited amounts for a fixed charge) and placement (checkouts, end-of-aisle and island/bin displays) promotion restrictions of HFSS discretionary foods (confectionery, sweet biscuits, crisps, savoury snacks, cakes, pastries, puddings and soft drinks with added sugar). Proposals related to the promotion of HFSS foods in any setting where food is sold to the public, including the out of home sector and wholesale outlets where sales are not only to trade. Proposals considered restricting retailers from being able to sell or lease display spaces for foods subject to the restrictions, and manufacturers and distributors from providing promotional or marketing material and from making arrangements for the display of foods subject to the restrictions. Consultation invited views on the type of promotions and food categories in scope, exemptions to the restrictions, approach to enforcement and support for implementation.

HFSS, high in fat, sugar or salt.

### Data analysis

Industry consultation responses were first categorised into types of industry actors based on information published on the Scottish Government consultation webpage. This included industry representative bodies, food and drink manufacturers, grocery retailers, out of home food retailers and advertising, media or broadcast organisations. All responses were uploaded to NVivo 20 (QSR International). Only relevant text related to unhealthy food and beverage retail promotion policies was coded (i.e. text discussing other topics included in the consultation 1 document such as food labelling and physical activity was not included in the analysis).

After reading all submissions in full for data familiarisation, initial inductive coding of 20 % of publicly available responses for each consultation (focusing on the longest submissions from all industry actors) was conducted separately by two members of the research team (CGD and SA). Categorisation of themes were discussed with several other members of the research team to achieve consensus in interpretation (KB, AC, JA, MW). This process identified that the PDM was an appropriate framework for analysis. The remaining documents were deductively coded by one member of the research team (CGD) based on the PDM. Quotations from consultation responses were used to illustrate findings.

## Results

The analysis included a total of 49 industry consultation responses related to restrictions on food retail marketing strategies using price and placement promotions (22 from c1 and 27 from c2) that were consented to be made publicly accessible. Industry submissions were predominantly from food and drink manufacturers, food retailers and industry representative bodies. Further details on the number of responses assessed across industry actor types can be found in Table 3.

**Table 3.** Number of responses submitted and made publicly accessible (%) by industry actors for ‘*A Healthier Future – Action and Ambitions on Diet, Activity and Healthy Weight’* 2017/18 (Consultation 1) and *‘Reducing Health Harms of Foods High in Fat, Sugar or Salt’* 2018/19 (Consultation 2)

Industry actors	Consultation 1					Consultation 2		
	*n*	Consent to publish		Provided response to questions related to retail promotion policies		*n*	Consent to publish	
		*n*	%	*n*	%		*n*	%
Industry representative bodies	18	17	94 %	14	78 %	13	13	100 %
Food and drink manufacturers	9	3	33 %	3	33 %	17	9	53 %
Out of home food retailers^*^	2	0		0		1	1	100 %
Grocery retailers	5	2	40 %	2	40 %	7	3	43 %
Advertising, media or broadcast organisations	4	3	75 %	0		1	1	100 %
Private sector weight management organisations	5	5	100 %	3	60 %	0	0	
Total	43	30	70 %	22	51 %	39	27	69 %

* For example: restaurants, fast food outlets and coffee shops.

Most industry responses supported the overall aim of the policies, which was to promote a healthy weight (c1) or improve the healthiness of diets and food environments (c2), but disagreed with the proposed policy to restrict unhealthy food promotions in retail settings or raised concerns and identified potential negative consequences associated with the policy. The exception was the weight management sector, which generally agreed with both the proposed policies and their stated aims.

### Discursive strategies

The arguments opposing the policy proposals were in line with the PDM and largely consistent across food industry actors, although specific concerns varied according to each actor’s specific commercial interests. Many of the identified discursive strategies overlapped across the PDM domains.

#### Unanticipated costs to the economy and society

A major concern raised by most industry actors was the potential negative impact of the proposed policies on the economy, particularly reduced industry profits and job losses. For example, the British Takeaway Campaign (industry representative body, c1) predicted that such policies would deliver a ‘*significant blow to a sector which is of vital importance to Scotland, having boosted the economy by almost £400 million in 2016’*. Similarly, Mackie’s of Scotland (manufacturer, c2) warned that the policies would ‘*likely lead to our chocolate factory closing’*. Industry responses frequently highlighted concerns about the potential misalignment of the proposed policy across different UK jurisdictions, suggesting this would complicate implementation. Some industry submissions also expressed concern that the policies would place an undue burden on local authorities and trading standards officers. The Food and Drink Federation Scotland (industry representative body, c2) stated that it would be absurd to ‘*end up with legislation that could see Trading Standards officials entering premises and issuing fixed penalty notices for e.g. chocolate bars on the counter of a small convenience store’*. A food manufacturing company, Border Biscuits (manufacturer, c2), questioned whether ‘*overstretched’* local authorities could ‘*realistically implement this’*. For small retailers, the Association of Convenience Stores (industry representative body, c2) argued that placement restrictions could impact the availability of essential community services like cash machines and post offices. They suggested that convenience retailers might need to change their layout and *‘make tough decisions about their product range or services that they offer and consider whether to remove the service or adapt their product range in order to be compliant with the regulations’.*

Industry submissions also highlighted other societal costs of the policy proposals, particularly their potential negative impact on consumers, such as increased economic pressure and restricted choice (even though the proposals did not restrict product availability in any way). For example, Boots UK (retailer, c2) stated that *‘consumers should be free to make their own choices, including whether to “treat” themselves as part of a healthy and balanced diet’* and that these proposals *‘may lead to unique inflationary pressure on customers baskets’*. The Snack, Nut and Crisp Manufacturers Association (industry representative body, c1) expressed concern that limiting promotions might impact household budgets, particularly for low-income families who rely on promotions and deals to save money during financially constrained times when ‘*household budgets continue to be squeezed’*. Some industry responses also expressed concern that retailers might raise prices to compensate for any downturn in sales or margins, further exacerbating economic pressure on consumers.

#### Unintended costs to public health

Additional anticipated costs of the policy were related to unintentional negative effects on public health, broadly defined. Industry submissions, especially those from food manufacturers, argued that the proposals were incompatible with existing measures aimed at improving population diets, such as nutritional reformulation. Businesses operating across the UK argued that these restrictions would negatively impact sales of new reformulated products aligned with the Public Health England reformulation programme (which does not apply in Scotland) like soft drinks and dairy products. Danone UK (manufacturer, c2) stated that *‘restricting promotions would make it more difficult for companies to rebalance their portfolio away from less healthy legacy products’*. Coca-Cola European Partners (manufacturer, c2) expressed concern that the proposed policies would undermine community activities and fundraising campaigns resulting from collaborations between manufacturers, charities and retailers. They highlighted examples such as soft drink promotions aimed at reducing alcohol consumption, as well as *‘valuable community initiatives, such as the Coca-Cola 5× 20 programme in Dundee’* (i.e. a corporate initiative to support female entrepreneurs).

Concern was also expressed regarding the potential negative impact the polices might have on the ability of vulnerable population groups, such as children and older adults, to meet their dietary requirements. The Food and Drink Federation Scotland (industry representative body, c2) claimed that ‘*11 % of teenage boys and 22 % of teenage girls are below the LRNI for calcium; restricting the opportunity to promote these desserts [ice cream and dairy desserts] could unintentionally lead to a further reduction in calcium intakes”* and that *”one of the suggested tips to prevent malnutrition in this group [the elderly] is to have a dessert after both lunch and dinner’.* Additional concerns were related to the impact of promotion restrictions on cultural relationships and connection with food and the potential stigma associated with discretionary food. Nestle UK & Ireland (manufacturer, c2) argued for an exemption for all seasonal discretionary products, emphasising that *“seasons such as Christmas and Easter are cultural events very closely linked to traditions”.*

Some submissions argued that price promotion restrictions could increase food waste as discounts are often used by retailers to incentivise the purchase of products at the end of their shelf life (even though restrictions would not apply to fresh products). One submission (Scottish Wholesale Association, industry representative body, c2) argued that the display of *‘high volume turnover items’* in prominent locations during festive seasons like Christmas ensures these products are easily accessible for safe restocking, suggesting that placement restrictions could compromise retail staff safety as a result of reduced options for product display. Other submissions highlighted that retailers might find ways to circumvent restrictions. For example, Boots UK (retailer, c2) suggested that *‘retailers may consider permanently reducing the price of discretionary food in order to maintain price comparison across England and Scotland’* and that this ‘*could result in increased consumption of these products’.*

#### Intended public health benefits

Submissions often contested the intended health benefits of the proposed policies, arguing that there was a lack of evidence that the policy will impact dietary behaviours, health outcomes or health inequalities. The British Soft Drinks Association (industry representative body, c2) cited a 2014 McKinsey study, claiming there was ‘*little evidence that initiatives such as restricting price promotions, marketing regulations or changes to store layouts would have any meaningful impact on obesity levels or health outcomes compared with the positive influence of reformulation and portion control strategies’.* Similarly, Scottish Bakers (industry representative body, c2) argued that ‘*marketing mechanisms listed in the consultation are designed to get consumers to switch within a category – restricting promotions won’t encourage them to switch to e.g. vegetables’*.

In addition, submissions often disputed the evidence related to the scope of the proposed policies. There was generally greater acceptance of restrictions on multi-buy promotions compared to temporary price promotions, and there was a perception that evidence on the influence of these promotional practices was lacking for out of home food retailers. Some submissions, particularly from food manufacturers and industry representative bodies like the Provision Trade Federation (c2), questioned the classification of certain products as unhealthy (e.g. reformulated soft drinks). Some submissions also highlighted product categories not included in the policy proposal. Mackie’s of Scotland (manufacturer, c2) argued that approximately 75 % of foods contributing to the nation’s obesity problem would not be covered by the policy, noting that *‘the proposed measures would have no restrictions on say a Lorne sausage, which has 20g of fat per slice!’*. Border biscuits (manufacturer, c2) drew attention to products with misleading health claims, arguing that *‘if people are consuming more of these types of products, thinking this is a healthier option, surely that’s more damaging to public health than knowingly having a higher sugar/fat/kcal treat?’.*

#### Expected costs to industry

In contrast to statements denying the influence of promotions on purchasing and dietary behaviours, many submissions argued that promotions are crucial for retailers and manufacturers to incentivise and attract customers, boost sales and sustain market competition. Submissions also underscored the ongoing challenges already faced by industry, particularly by small stores and businesses that have already been targeted by other regulatory measures.

Paterson Arran (manufacturer, c2) claimed that *‘banning the promotional display of our products would reduce our sales by between £0·5M and £1M per annum and reduce our operating profit by up to 30 %’.* The Scottish Wholesale Association (industry representative body, c2) argued that in some cases the *‘entire product range will be affected by these disproportionate proposals putting jobs and future investment at risk’.* Their wholesale members claimed that 30 % of their discretionary food sales are made through promotions and that some independent retailers rely on these promotions to remain operational. Submissions often emphasised that the proposed measures would add to the existing financial burden faced by the industry, including market, regulatory and taxation pressures. Danone (manufacturer, c2) noted that *‘companies in the food and drink sector are facing a number of challenges specific to its sector, including reformulation, the soft drinks industry levy, a potential packaging tax, as well as unresolved issues regarding the supply chain and talent in the sector as part of the Brexit’.* The Association of Licensed Multiple Retailers (industry representative body, c1) argued that introducing further measures on soft drinks would be *‘unfair and unjustified’* until the impacts of the soft drinks levy are fully assessed, cautioning against placing additional burdens on products that have already made significant sugar reductions.

Promotions were also considered important for building excitement around new products, with industry responses suggesting that the proposed policies would negatively impact product innovation and investment in novel technologies. Paterson Arran (manufacturer, c2) anticipated that this would *‘erode our sales and be a significant disincentive to the major retailers continuing to list our products’.* The British Soft Drinks Association (industry representative body, c2) echoed this view, stating that *‘promotions strengthen competition and ensure that consumers are provided with a choice of products at competitive prices’.* Submissions from retailers and manufacturers emphasised the role of promotional displays in helping retailers to maximise space. They argued that placement promotions, such as end of aisle, checkouts and island/bin displays, provide additional selling space and that the proposed restrictions would limit the range of available products. This was seen as particularly challenging for smaller stores, as noted by the Scottish Grocers Federation (industry representative body, c2): ‘*To stay in business convenience retailers have been forced to maximise every inch of selling space within the store. Smaller stores have little option but to use end of aisles and checkouts to display confectionery and similar product categories’.* The Scottish Wholesale Association (industry representative body, c2) also highlighted the challenges of reduced display options, particularly during festive seasons when *‘most wholesalers and their retail customers only have the option of displaying those [festive] products on rack-ends (gondola ends), floor displays or front of checkouts’*. Promotional displays were also considered key tools for establishing brand reputation and awareness.

Submissions from small and local retailers and manufacturers argued that the proposed restrictions would disproportionately affect them compared to larger businesses given their differing resources. The Association of Convenience Stores (industry representative body, c2) expressed concerned that ‘*larger retailers may have more flexibility to adapt to the regulations than smaller retailers, for example, using alternative promotional activity such as “everyday low prices’.*

### Instrumental strategies

Information management and direct involvement and influence over policy makers were identified as the main tactics for the presentation and dissemination of the discursive strategies (arguments) throughout the consultation responses.

#### Information management

Industry submissions relied on anecdotal evidence, customer feedback and unsubstantiated claims to question and undermine policy support. For example, Mackie’s of Scotland (manufacturer, c2) claimed that *‘approximately 75 % of food which are contributing to the nation’s obesity problem would not be covered’* without providing any supporting evidence. Similarly, many submissions, particularly from food manufacturers, cherry-picked data to support their claims. Coca Cola European Partners (manufacturer, c2) stated that they *‘are focusing on the interventions that all the available evidence shows work best for consumers – such as reformulation, portion control and low calorie innovation’.* Submission often used specious claims (i.e. superficially plausible, but actually wrong and therefore deliberately misleading). For example, Coca-Cola European Partners (manufacturer, c2) stated that the policies would *‘create unnecessary complexity and additional costs for manufacturers and retailers for those products not made exclusively for the Scottish market*’.

Submissions also aligned arguments with their own interests and shifted focus to other public health concerns, often using evidence from unidentified sources. The Scottish Grocers Federation (industry representative body, c2) claimed that *‘data from 2016 suggests that 19 % of consumers regularly purchase treats and snacks in convenience stores, while 46 % of consumers regularly buy treats and snacks in a supermarket’.* Food and Drink Federation Scotland (industry representative body, c2) stated that *‘there are approximately 3 million people in the UK who are malnourished or at risk of malnutrition’,* reasoning that ‘*although the policy focus is on decreasing obesity, we believe consideration should also be given to malnutrition, particularly in the elderly’*. Some submissions positioned the industry as a trusted source of public sentiment, emphasising the supposed unpopularity of the policy. Asda (grocery retailer, c1) noted that *‘customer insight shows that a ban on price promotions is not supported’,* arguing that their customers value *‘the opportunity to shop around based on the deals on offer, particularly during key seasonal periods’* and that they *‘see price competition between retailers as a key contributor to keeping down the cost of living’.*

Finally, signs of a coordinated approach among industry actors were evident, with submissions expressing mutual support and consistent arguments. Many shared their investment in and commitment to existing initiatives to promote healthy diets, while collectively advocating a preference for restricting certain type of promotions like multi-buys. For instance, Potato Processors Limited (industry representative body, c2) declared that they *‘support FDF [Food and Drink Federation] Scotland’s view that reformulation should be rewarded and any promotional restrictions must not undermine work undertaken by companies’.*

#### Direct involvement in and influence over the policy process

Most manufacturers and retailers sought to increase their involvement in the policy process, proposing alternative policy recommendations and in-person consultations. Border biscuits (manufacturer, c2) demanded that the proposal *‘should not change without further consultation’.* Tesco (grocery retailer, c2) acknowledged logistical in-store complexities and offered to ‘*host a team from the Scottish Government at one of our stores to explain how we currently arrange products and explore ideas for making constructive changes in the future’*.

Many responses suggested that the proposals were redundant, recommending industry-preferred solutions such as educational initiatives and nutrient reformulation, which they claimed would be more beneficial. Proposed alternatives tended to be vague (i.e. ‘holistic’ interventions), emphasising individual responsibility and promotion of healthy foods. Scottish Slimmers (private sector weight management organisation, c1) argued that *‘as a nation, we need to place more focus on promoting and providing healthy food’.* McDonald’s (out of home food retailer, c2) stated that ‘*the best way to encourage better choices by customers is to provide them with the information they require to make informed decisions’*. Others stated that *‘a more nuanced approach’* was required, one *‘that maintains an incentive to reformulate existing products, and to bring new products onto the market that are nutritionally improved’* (Nestle UK & Ireland, manufacturer, c2). The Scottish Wholesale Association (industry representative body, c2) advised the government ‘*to remove the proposals considered within this consultation and work towards a unified UK wide approach’.*

Submissions often attempted to shape public policy development in alignment with their interests, frequently advocating for less comprehensive policy designs that favour their own product sales. Many recommended excluding certain types of price promotions, such as meal deals, which they argued were less likely to encourage unhealthy dietary behaviours. Several submissions requested the exclusion of their own ‘healthier’ products such as reformulated soft drinks, fruit juices and dairy. Others anticipated that their ‘specialised nutrition’ products, such as infant and toddler foods, would be excluded from the policy scope (Danone, manufacturer, c2).

## Discussion

This study documents the discursive and instrumental strategies used by industry actors in response to a public consultation process for a government-led policy proposal to restrict unhealthy food retail promotions. Based on the nature of consultations, discursive strategies were more likely to be captured; however, instrumental strategies were also identified when possible. Whilst we only evaluated one of the many ways that industry can influence government policy making, our findings can be used by other jurisdictions across the world to prepare for opposition when pursuing a similar policy.

Industry consultation submissions in Scotland raised a range of concerns about the policy proposals, including potential negative impacts on the nation’s economy and customer budgets, despite the consultations occurring before the UK’s inflationary pressures and cost-of-living crisis^(31)^. Submissions also highlighted a perceived misalignment with existing efforts to promote healthier diets like nutritional reformulation, disregarding that HFSS-based retail marketing restrictions are partly aimed at incentivising reformulation. Concerns were raised about the enforceability of the policy, with claims that industry actors would circumvent restrictions by adopting strategies such as ‘everyday low prices’. Discursive strategies were closely aligned with the PDM, although submissions did not always downplay the potential direct costs to their own industry. While responses highlighted the disproportionate disadvantages faced by small retailers and burdens placed on businesses already affected by regulatory measures, they also acknowledged that promotion restrictions would affect overall retail sales. Industry responses questioned the evidence supporting the policy’s effectiveness, portraying awareness of and commitment to academic standards for evidence use. Simultaneously, industry submissions often included unsubstantiated and exaggerated claims to support their arguments, demonstrating double standards through an inconsistent adoption of evidence criteria. Other instrumental strategies included inviting government representatives to grocery stores in an attempt to subvert the formal public consultation process, requesting further consultations to potentially delay policy implementation, and diverting attention away from the proposals’ intended purpose.

Our findings are consistent with a recent study that explored how hypothetical policies targeting price promotions in supermarkets are perceived by food industry actors in Australia^(32)^. Another qualitative study explored the perceptions of different actors (consumers, manufacturers, retailers, enforcement officers and academic and charitable health representatives) regarding UK legislation restricting the promotion of HFSS products in prominent retail locations after the policy had been announced, but not enacted^(33)^. Although these actors generally supported the policy, they identified several recommendations including ring-fenced resources for local authorities to ensure compliance and legal and financial support for smaller businesses. The legislation later enacted in England excludes small and specialist retailers, with the government response recognising the additional burden that implementation would place on these businesses^(15)^. Public health researchers in the UK have argued that this gap in policy coverage may potentially widen inequalities, as populations known to have poorer diets (e.g. young people, older adults and socio-economically disadvantaged families) often rely on small convenience stores to purchase food^(34)^.

Our findings are also in line with the broader literature demonstrating the arguments used by the food industry to oppose public health policies^(25–27)^. In our study, industry responses claimed that existing measures were sufficient, and that further government intervention was unnecessary. The narrative of policy redundancy is a common strategy used globally to oppose food policies, including in the UK during the development of the soft drinks levy^(35)^. Although evidence underscores the importance of implementing several policies to achieve the greatest impact on population diets and health^(36)^, industry initially framed the soft drinks levy as incompatible with other measures such as reformulation and advertising codes. However, evidence since the levy’s implementation shows that soft drink companies have reformulated their products to a lower sugar content in order to avoid the levy^(37)^ with no significant adverse impact on businesses^(38)^. Other narratives identified in industry responses to the UK consultation on the soft drinks levy, such as unintended consequences of the levy and the responsibility of government to industry over health^(35)^, also align with our findings. Evidence suggests that the industry narrative regarding SSB taxes changed over time based on the stage of the policy process (i.e. from outright opposition to attempting to delay or weaken the policy after its announcement, and then adapting after its implementation)^(27)^.

Most industry submissions repeatedly highlighted a lack of evidence for the effectiveness of the proposed policies on dietary behaviour and health outcomes, a common argument also seen in response to other public health policies^(27,35)^. This is despite robust evidence showing that customers are highly responsive to price promotions, particularly for unhealthy food like SSBs^(11–13)^. Both temporary price reductions and multi-buys lead to higher purchase volumes^(10,39)^, which can result in stockpiling and increased overall consumption of promoted products^(39)^, particularly among regular consumers^(10)^. It can also be argued that price promotions can undermine the effectiveness of public health policies like SSB taxes^(5,6)^. Following the Oakland SSB tax, the magnitude of price promotions on SSBs increased, countering the intended impact of the tax^(5)^. In Scotland, the implementation of a 2011 ban on alcohol multi-buy promotions led to an increase in temporary price discounts, with no significant effect on the overall volume of alcohol purchased^(40)^. This may help explain why industry consultation submissions showed a preference for restrictions only applying to some types of price promotions and highlights the importance of a comprehensive policy design. Similarly, for placement promotions, there is evidence that companies are exploiting gaps in the legislation implemented across England by promoting HFSS products in mid-aisle displays^(41)^. These adaptations to regulations are expected from commercial actors residing in a complex adaptive system that will respond to external stimuli to maintain current profits and growth by alternative means^(6)^. Robust policy evaluation is critical to identify and address these adaptations.

Another key argument against the proposed policy was the perceived financial burden on consumers. Evidence suggests that while price promotions make products cheaper, they also encourage consumers to purchase higher volumes than they would without the promotion^(42)^. Crucially, the proposed legislation only seeks to limit price promotions on unhealthy foods. HFSS products are more likely to be purchased on price promotion than healthier food^(9)^, and while promotions may offer short-term financial benefits to low-income families, they do not account for the long-term health costs associated with increased consumption of unhealthy food^(2)^. A study exploring women’s perceptions of restrictions on HFSS product promotions in England revealed that most participants acknowledged that the high price of healthier foods could limit the benefits for low-income families^(43)^. Industry submissions also argued that promotions are essential for sustainable market competition. It is important to note that these marketing strategies currently represent a competitive advantage for transnational corporations, which have greater power to negotiate and benefit from product placement and price promotions^(44)^. Evaluations of the implementation of this policy in England will be important to determine if it results in more price promotions for healthy food, which could help make a healthy diet more affordable.

Industry submissions frequently relied on unidentified or inaccessible sources to support unsubstantiated statements, with many arguments underpinned by specious claims. As other studies have noted, this underscores the need to critically appraise the industry’s use of evidence to enhance transparency and accountability^(27,45)^. This may involve providing guidance on what constitutes appropriate evidence for consultation submissions and/or training of government representatives to assess evidence quality when synthesising responses. Most manufacturers and retailers sought to increase their involvement in the policy and decision-making process, setting expectations for further consultation on policy design and proposing direct interaction with policy makers. Similar strategies, including the request of additional evidence of policy effectiveness, were used in France during the development of a new front-of-pack nutrition labelling system, contributing to policy delays^(25)^. Evidence shows that industry actors often claim more authority (e.g. providing technical advice, contesting the expertise of government) compared with technical experts, civil society and government actors, which may enhance their perceived legitimacy and influence over policy development and implementation^(46)^.

Strengths of this study include the analysis of a large number of industry responses, the initial independent inductive coding conducted by two researchers and the use of a well-established framework for describing corporate political activity. The PDM strategies are generalisable and can be applied to assess industry activity across different policies and jurisdictions, allowing comparison across studies. Our findings are likely to be relevant for other jurisdictions considering implementing food retail marketing policies, except for specific arguments related to the UK political and regulatory context. There are also some limitations to consider. Our analysis assessed publicly available industry submissions from two consultations and did not include submissions from the latest Scottish Government consultations on retail promotion restrictions, as these were not published at the time of analysis. We had no access to responses that did not consent to be published, which may differ from publicly available responses (e.g. may include confidential data or more direct evidence of instrumental strategies such as reference to meetings). Given the similarity of corporate playbook strategies^(23)^, our findings are likely to be applicable to all consultation responses, although instrumental strategies may be underrepresented. We also had no access to details of other corporate political activities beyond consultation submissions (e.g. expenditure on lobbying, meetings), which are also commonly used by industry actors^(23)^. Consequently, our findings likely reveal just a part of the picture of the industry’s political activity. Additionally, it is worth noting that whether the discursive and instrumental strategies used by industry in the analysed submissions have influenced policy design and development in Scotland remains unknown.

## Implications and future research

Since the first two consultations, the Scottish Government has commissioned several studies to further inform the policy and opened two additional consultations^(20,21)^. Analysing the final policy design could give further insight into the influence of industry’s political activity, which could be assessed more comprehensively through increased lobbying transparency. Defining when, where and how different industry actors are allowed to be involved in the policymaking process could be helpful to limit the influence of pre-existing power structures^(47)^. Although we did not analyse non-industry submissions, such as those from academic, public health and third sector organisations, these actors also play an important role in framing and influencing food environment policies. A study assessing advocacy strategies and congruence in the UK press discourse during the development of two pricing policies suggested that policy opponents had greater cross-sector collaboration than proponents^(48)^. Other studies have highlighted the need for developing and testing counter-framing messages that successfully offset the prevailing industry-friendly framings that reflect corporate rather than public health interests^(49)^. Future research should analyse responses from non-industry actors to understand how their discourse differs from industry in consultation submissions.

Our findings can be used to support policy proponents, including public health actors, to anticipate industry arguments on novel policy proposals targeting unhealthy food retail promotions. Whereas the use of arguments can be countered by non-industry actors and evaluated by civil servants, further action is required to address instrumental strategies, including robust management of conflicts of interest and establishment of guidance on using evidence in consultation responses. Examples of mechanisms for addressing instrumental strategies include increased transparency and disclosure of all forms of corporate political practices as well as dedicated personnel to review the evidence in consultation submissions according to previously defined guidelines^(50)^.

## Conclusion

Our findings add to a growing body of evidence on strategies used by the food industry to avoid public health regulations that may reduce their profits from sales of unhealthy foods. These findings can be used to anticipate and address industry responses to help advance healthy food retail policies in the broader public interest. Public health actors may counter industry arguments through targeted communication strategies that reinforce the intended purpose and scope of proposals, while government action must ensure robust management of conflicts of interest and establishment of guidance for the use of supporting evidence as part of the public health policy process.
